# Association of military life experiences and health indicators among military spouses

**DOI:** 10.1186/s12889-019-7804-z

**Published:** 2019-11-12

**Authors:** Nida H. Corry, Sharmini Radakrishnan, Christianna S. Williams, Alicia C. Sparks, Kelly A. Woodall, John A. Fairbank, Valerie A. Stander

**Affiliations:** 1 Abt Associates, Central Park West, Suite 210, 5001 South Miami Boulevard, Durham, NC 27703 USA; 2 Abt Associates, 10 Fawcett Street, Cambridge, MA 02138 USA; 3 Abt Associates, 6130 Executive Boulevard, Rockville, MD 20852 USA; 4 The Henry M. Jackson Foundation for the Advancement of Military Medicine, 6720A Rockledge Drive, Suite 100, Bethesda, MD 20817 USA; 5 Mid-Atlantic (VISN 6) Mental Illness Research, Education and Clinical Center, Durham VA Health Care System, 3022 Croasdaile Dr, Durham, NC 27705 USA; 6 Department of Psychiatry and Behavioral Sciences, Duke University School of Medicine, 1121 West Chapel Hill Street, Suite 201, Durham, NC 27701 USA; 7 Military Population Health Department, Naval Health Research Center, 140 Sylvester Road, San Diego, CA 92106 USA

**Keywords:** Military, Service member, Military spouse, Health behaviors, Healthy People 2020

## Abstract

**Background:**

The health and well-being of military spouses directly contribute to a robust military force by enabling the spouse to better support the active duty member’s career. In order to understand the overall health and well-being of military spouses, we assessed health indicators among military spouses using the Healthy People 2020 framework and examined associations of these health indicators with military experiences and psychosocial factors.

**Methods:**

Using data from the Millennium Cohort Family Study, a U.S. Department of Defense-sponsored survey of 9872 spouses of service members with 2–5 years of military service, we examined attainment of Healthy People 2020 goals for spouses and service members, including healthy weight, exercise, sleep, and alcohol and tobacco use. Multivariable logistic regression models assessed associations of spouse health indicators with stressful military life experiences and social support, adjusting for demographics and military descriptors. The spousal survey was administered nationwide in 2011.

**Results:**

The majority of military spouses met each health goal assessed. However, less than half met the healthy weight and the strength training goals. Reporting greater perceived family support from the military was associated with better behavioral health outcomes, while having no one to turn to for support was associated with poorer outcomes. Using the Healthy People 2020 objectives as a framework for identifying key health behaviors and benchmarks, this study identified factors, including military-specific experiences, that may contribute to physical health behaviors and outcomes among military spouses. With respect to demographic characteristics, the findings are consistent with other literature that women are more likely to refrain from risky substance use and that greater education is associated with better overall health outcomes.

**Conclusions:**

Findings suggest that enhanced social and military support and tailored programming for military spouses may improve health outcomes and contribute to the well-being of military couples. Such programming could also bolster force readiness and retention.

## Background

Promoting healthy behaviors and outcomes has long been a priority for the United States military, and since 1986, the Department of Defense (DoD) has emphasized its Health Promotion and Disease Prevention directive, which provides health guidance and encourages healthy living goals among military personnel and their beneficiaries [1]. Health behaviors that put individuals at risk of physical and social consequences are alarmingly prevalent among service members, particularly those involving substance use [2–4]. One DoD study found that 39.6% of all active duty current drinkers reported binge drinking in the past month; and 24.5% of active duty service members reported cigarette use in the past month; additionally, 51.2% of active duty personnel were classified as overweight, despite the military’s high physical health standards [3]. Poor health behaviors negatively affect not only the individual, but also their families and broader society, causing an increase in missed days from work and health care costs [5]. The health and well-being of military spouses also directly contribute to a robust military force by enabling the spouse to better support the active duty member’s career [6] and have significant health care cost implications. A study of TRICARE beneficiaries (dependents of active duty personnel, military retirees, and dependents of military retirees) found that each year DoD spends approximately $2.1 billion for medical care associated with obesity, alcohol use, and tobacco use [2].

There is less data available regarding the health of military spouses compared to service members; however, some studies shed light on potential health issues. A 2012 presentation found that one in five Army active duty spouses are overweight, one third are obese [7] and studies suggest that service member deployment is not a predictor of spouses’ overweight or obesity [8, 9]. Approximately 8.2% of military spouses married to service members with 2–5 years of experience reported alcohol misuse [10] and unhealthy alcohol use among military spouses was associated with feeling bothered by communication about the service member’s deployment experiences as well as the spouse feeling stressed by a combat-related deployment or duty assignment [11].

In an effort to combat health disparities and to improve health outcomes for all Americans, the U.S. Department of Health and Human Services launched a health promotion program and evaluation measure called the Healthy People initiative [12]. The Healthy People 2010 initiative (HP2010) analyzed 28 different focus areas containing 467 measurable objectives of physical health from 2000 to 2010, and these objectives were updated for Healthy People 2020 (HP2020) [13]. Although research on the U.S. population shows that particular population groups are disproportionally affected by poor health outcomes and have less access to preventive care (e.g., individuals living below the poverty line, individuals in rural populations, and racial/ethnic/sexual minorities) [14–18], there has been very little research investigating the relative achievement of Healthy People objectives among military populations, and particularly military spouses. In a 2006 study, self-reported service members’ health behaviors met or exceeded 7 of the 19 HP2010 objectives assessed, including those related to obesity and exercise [19]. Kress and colleagues assessed HP2010 objectives among military retirees and their spouses and found that fewer retirees and beneficiaries met obesity, exercise, substance use, and healthy eating objectives than national target percentages [20]. Being male, having less than a college degree, and less-than-excellent self-reported health status were associated with a lower likelihood of meeting the objectives [20, 21].

Despite DoD’s commitment to the health and well-being of all members of the military community, military spouses may be at greater risk for poor health behaviors than their military partners or civilian counterparts. Military spouses do not have the same incentives and structure to help them maintain their health, yet they are exposed to many stressors unique to military life, such as relocation and deployment, that may challenge healthy living [22, 23]. Additionally, access to military health promotion programs and support systems that have been shown to reduce poor health behaviors [24–26] is uneven, particularly for certain subgroups, such as male and minority spouses and spouses of those serving in National Guard and Reserve components.

In order to understand the overall health and well-being of military spouses, the current study aimed to investigate various health behaviors and indices, including weight, exercise, sleep, and substance use, using data from the Millennium Cohort Family Study (henceforth referred to as the Family Study), which is a probability-based cohort [27–29]. We have used the HP2020 goals framework to assess health indicators among military spouses and assess associations between these health indicators, operationalized as attainment of HP2020 goals in several domains, and sociodemographic characteristics, military experiences, and psychosocial factors.

## Methods

### Sample design and study participants

This analysis used the Family Study baseline sample, which consists of 9872 service member/spouse dyads. The service members are participants in the Millennium Cohort Study who were married and had 2 to 5 years of military service as of 2011. Married and female service members were oversampled in the Millennium Cohort Study to ensure that male spouses of female service members were adequately represented in the Family Study. Spouses of participating service members were then recruited in 2012 to complete the dyads. The sample is unique in that it includes a representative sample of young military couples, from all service branches and components (active duty, military Reserve, and National Guard participants).

The Family Study methods are described in more detail elsewhere [27–29]. The Family Study was overseen and approved by the Naval Health Research Center’s Institutional Review Board (Protocol 2000.0007) and the Office of Management and Budget (approval number 0720–0029). Written or electronic informed consent was obtained from all participants.

### Measures

Service members and their spouses independently completed surveys addressing various aspects of physical and mental health as well as their health behaviors. Additionally, participants provided permission to merge their survey responses with archival data on their military personnel and medical treatment records. Although the analyses for this paper focused primarily on the spouses’ survey responses regarding health, we did include several predictors and covariates from Millennium Cohort research program data resources, described below.

#### Health outcomes

Six dichotomous indicators were used to identify spouses who met the HP2020 goals with respect to healthy weight (body mass index; BMI), aerobic exercise, strength training, sleep, alcohol use (risky drinking), and tobacco use (current smoking). The criterion definitions for each of the goals map on closely to the respective HP2020 goals and are detailed in Table 1. For aerobic exercise and strength training goals, spouses who stated that they did not or were unable to physically engage in these types of exercise were coded as not meeting the goals. Service member health indicators were measured in the same manner as the corresponding spouse health measures.

**Table 1:** Definitions of Health People 2020 Goals

Domain	HP2020 Individual Goal^a^	Assessment of Goal for Spouses and Service Members Using Family Study Data
Weight/BMI	Attain a healthy weight for height: body mass index (BMI), 18.5–25 kg/m^2^	Self-reported weight and height, calculated to a BMI in healthy range based on self-reported height and weight (yes/no)
Aerobic exercise	Engage in aerobic physical activity of at least moderate intensity for at least 150 min/week, or of vigorous intensity for 75 min/week, or an equivalent combination (calculated as the sum of moderate intensity minutes and 2 times the vigorous intensity minutes)	Self-reported aerobic exercise minutes in healthy range (yes/no); participants were asked about frequency and duration of both moderate and vigorous aerobic activity in a typical week
Strength training	Perform muscle strengthening activities on 2 or more days of the week	Self-reported strength training days per week in a typical week in healthy range (yes/no)
Sleep	Obtain adequate daily sleep – 8 or more hours for those aged 18 to 21 years and 7 or more hours for those aged 22 years and over	Self-reported hours of sleep in an average 24-h period over the past month in healthy range (yes/no)
Alcohol use	Refrain from risky alcohol consumption, either in the form of heavy drinking or binge drinking, in the past 30 days	Risky drinking, defined as self-reported heavy drinking in the past week or binge drinking in the past year (yes/no)
	Heavy drinking, defined as consuming more than 2 drinks per day on average for men or more than 1 drink per day on average for women in the past 30 days.	Heavy drinking, defined as consuming 14 or more drinks in the past week for men or 7 or more drinks in the past week for women.
	Binge drinking, defined as consuming 5 or more drinks for men or 4 or more drinks for women at the same time or within a couple of hours of each other in the past 30 days.	Binge drinking, defined as consuming 5 or more drinks on 1 occasion for men or 4 or more drinks on 1 occasion for women in the past year.
Tobacco use	Refrain from smoking cigarettes	Current nonsmoker based on self-report; respondent is classified a current smoker if they have smoked at least 100 cigarettes in their lifetime and also smoked cigarettes in the past year (yes/no)

^a^Healthy People 2020

#### Social support and military stress experiences

Measures of military and social support were included as independent variables. Military support was measured with 2 items: *perceived military support for the spouse and their family* and *perceived military support for the service member*. These are both ordinal variables, where 0 = “Poor”, 1 = “Fair”, 2 = “Good”, 3 = “Very good”, and 4 = “Excellent.” Four ordinal items addressed social support for the spouse respondent. One item from the Patient Health Questionnaire (*the degree to which you are bothered by not having someone to turn to in the last 4 weeks*) had 3 categories (1 = “Not bothered”, 2 = “Bothered a little”, 3 = “Bothered a lot”). Spouses were asked 3 additional questions about social support (*having someone to turn to when dealing with personal problems, having someone to tell you honestly how you are handling problems,* and *how well family and friends have supported you in the last 4 weeks*) on 5-point scales (0 = “Strongly disagree” or “Not at all” to 4 = “Strongly agree” or “Extremely”).

Other independent variables included 4 aspects of the stress of military life: deployment stress, injury stress, family stress, and stress resulting from one or more permanent change of station (PCS) moves. For each of first three domains: deployment (e.g., a combat-related deployment or duty assignment for your spouse), injury (e.g., combat-related injury to your spouse), and family stress (e.g., difficulty balancing demands of family life and your spouse’s military duties), the mean of three items was calculated [27]. Each of the items was scored from 0 to 4 (0 = *never experienced*, 1 = *not at all stressful*, 2 = *slightly stressful*, 3 = *moderately stressful*, 4 = *very stressful*). The deployments and injuries referred to by these items were experienced by the service member, not the spouse. A single item assessed the perceived stress of PCS moves with the same 0–4 scoring as the other military stress items.

#### Covariates

In addition to the independent variables listed above, we included spouse sociodemographics and several service member’s military characteristics. Spouses’ self-reported characteristics included gender, age, race and ethnicity, educational attainment, employment status, annual household income, number of children, and prior or current military service. Participants were asked to select from the following race/ethnicity options: White non-Hispanic, Black non-Hispanic, Asian/Pacific Islander, Hispanic, Native American, or Other. For analytic purposes, participant were categorized as White non-Hispanic, Black non-Hispanic, or Other. Service member military characteristics included pay grade (officer vs enlisted), branch of service (Army, Air Force, Navy, Marine Corps, Coast Guard), and component (active duty vs Reserve or National Guard). These data were obtained from administrative records provided by the Defense Manpower Data Center.

### Analyses

After generating descriptive statistics on the demographics and measures of stress and support, we examined attainment of six HP2020 goals for the spouses and service members by calculating the proportions meeting each goal. Additionally, we examined the concordance of spouse-service member pairs with respect to goal attainment in each domain. Finally, we estimated a multivariable logistic regression model for each spouse health outcome to investigate its unique associations with military life experiences and social support, as well as with the demographic and military characteristics. All social and military support independent variables were used as continuous measures in the models. Adjusted odds ratios can be interpreted as the relative change in odds of a particular spouse meeting a health indicator associated with each additional military stressor experienced or a 1-unit increase in perceived social support. All analyses were weighted to account for the sample design and nonresponse; these weights allow the findings to be generalized to the population of married spouses of service members with 2 to 5 years of military experience [29].

## Results

### Population description

Most spouses were female (86%) and between 25 and 34 (62.1%) years of age (Table 2). More than 70% were White and 53.2% had some college experience or an associate degree. Approximately two thirds (63%) had at least one child. The vast majority of spouses (80.6%) had no history of military service; 9.4% were currently serving in the military. Half of the spouses’ service member partners served in the Army, followed by 17.4% in the Air Force, 15.3% in the Marine Corps, 14.2% in the Navy, and 2.8% in the Coast Guard.

**Table 2:** Description of Family Study Cohort (2012) (*N* = 9872)

Characteristic^a^	N^b^	Prevalence, (95% Confidence Interval)^c^
Spouse Demographic Characteristics		
Gender		
Male	1273	14.0 (12.9–15.1)
Female	8599	86.0 (84.9–87.1)
Age, years		
17–24	2269	28.3 (27–29.6)
25–34	6391	62.1 (60.7–63.4)
35+	1208	9.6 (8.9–10.4)
Race/ethnicity		
White, non-Hispanic	7688	71.0 (69.6–72.4)
Black, non-Hispanic	411	8.1 (7.1–9.1)
Other	1720	20.9 (19.7–22.1)
Educational attainment		
High school graduate, GED or less	1278	16.4 (15.3–17.5)
Some college/associate degree	4569	53.2 (51.8–54.5)
Bachelor’s degree or higher	4003	30.4 (29.2–31.6)
Number of children		
0	3623	36.8 (35.4–38.1)
1	2799	29.3 (28–30.5)
2+	2200	34.0 (32.7–35.3)
Employment status		
Full-time	3433	35.1 (33.8–36.4)
Part-time	1278	12.8 (11.9–13.7)
Unemployed	1380	16.3 (15.2–17.4)
Homemaker/student	3754	35.8 (34.5–37.1)
Household income		
Less than $25,000	1136	15.0 (14–16.1)
$25,000–$49,999	4188	50.3 (48.9–51.6)
$50,000–$74,999	2321	21.5 (20.4–22.6)
$75,000 or more	2091	13.2 (12.4–14)
Military service		
Never	8107	80.6 (79.4–81.7)
Former	840	10.1 (9.2–11)
Current	916	9.4 (8.5–10.2)
Service member military characteristics		
Service component		
Active duty	7685	78.9 (77.8–80)
Reserve/National Guard	2187	21.1 (20–22.2)
Pay grade		
Enlisted	7453	91.0 (90.5–91.5)
Warrant or commissioned officer	2419	9.0 (8.5–9.5)
Service branch		
Army	4563	50.3 (48.9–51.7)
Navy	1407	14.2 (13.3–15.2)
Marine Corps	932	15.3 (14.2–16.4)
Air Force	2694	17.4 (16.4–18.3)
Coast Guard	276	2.8 (2.4–3.2)
Social and military support		
Spouse is bothered by having no one to turn to		
Not bothered (1)^d^	7122	70.1 (68.8–71.4)
Bothered a little (2)	1783	19.3 (18.2–20.4)
Bothered a lot (3)	836	10.6 (9.6–11.5)
Mean (SE)	1.40	(0.10)
Spouse has someone to turn to for suggestions on dealing with personal problems		
Strongly disagree (0)	280	3.4 (2.8–3.9)
Disagree (1)	502	5.6 (5–6.3)
Neither agree nor disagree (2)	956	11.1 (10.2–12)
Agree (3)	4401	43.7 (42.3–45)
Strongly agree (4)	3598	36.2 (34.9–37.5)
Mean (SE)	3.04	(0.01)
Spouse has someone to tell them honestly how they’re handling problems		
Strongly disagree (0)	307	3.5 (3–4)
Disagree (1)	518	6.0 (5.3–6.8)
Neither agree nor disagree (2)	984	10.6 (9.7–11.5)
Agree (3)	4346	43.4 (42.1–44.8)
Strongly agree (4)	3575	36.4 (35.1–37.8)
Mean (SE)	3.03	(0.01)
Support from family or friends		
Not at all (0)	429	5.1 (4.5–5.8)
A little bit (1)	1080	11.7 (10.8–12.6)
Moderately (2)	1625	17.0 (15.9–18)
Quite a bit (3)	3183	30.6 (29.4–31.9)
Extremely (4)	3462	35.5 (34.2–36.8)
Mean (SE)	2.80	(0.02)
Military efforts to help spouse and family		
Poor (0)	1762	19.8 (18.7–20.9)
Fair (1)	2400	25.2 (24–26.4)
Good (2)	2959	29.2 (28–30.4)
Very good (3)	1627	15.1 (14.1–16)
Excellent (4)	982	10.7 (9.8–11.6)
Mean (SE)	1.72	(0.02)
Military efforts to help service member		
Poor (0)	1564	18.2 (17.1–19.3)
Fair (1)	2176	22.8 (21.6–24)
Good (2)	2966	29.7 (28.4–30.9)
Very good (3)	1920	17.7 (16.7–18.7)
Excellent (4)	1102	11.6 (10.7–12.5)
Mean (SE):	1.82	(0.02)
Military stressors		
Deployment stress (mean of 3 items each ranging from 0 to 4)	9679	
Mean (SE)	1.6060	(0.02)
Injury stress (mean of 3 items each ranging from 0 to 4)	9679	
Mean (SE)	0.6767	(0.02)
Family stress (mean of 3 items each ranging from 0 to 4)	9680	
Mean (SE)	1.6767	(0.02)
Permanent change of station stress		
Never experienced (0)	4752	49.3 (47.9–50.7)
Not at all stressful (1)	1098	12.3 (11.3–13.2)
Slightly stressful (2)	1357	13.6 (12.7–14.6)
Moderately stressful (3)	1279	12.6 (11.7–13.5)
Very stressful (4)	1097	12.1 (11.2–13.1)
Mean (SE)	1.26	(0.02)

*Note*. *BMI* body mass index, *GED* general equivalency diploma

^a^All characteristics refer to the Family Study (spouse) respondent unless otherwise indicated.

^b^N’s are unweighted and do not consistently add up to 9872 because of missing data. The number missing ranges from 0 (gender and service member military characteristics) to 289 (permanent change of station stress)

^c^Prevalence estimates are weighted to represent the target population. The unweighted percentage can be calculated using the N for a given row as the numerator and the sum of the N’s for a given characteristic as the denominator.

^d^Numbers in parentheticals indicate the coding used in regression models.

#### Healthy People 2020 objectives

**Fig. 1:**
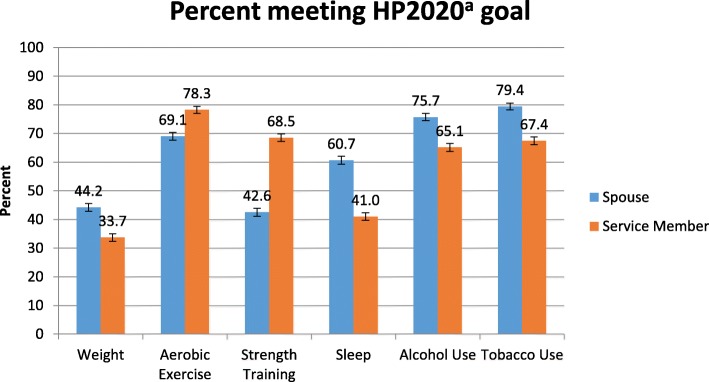
Percent meeting HP2020^a^ goal. Caption: HP2020 Goal Attainment for Family Study Spouses and Service Members. Refer to Table 1 for HP2020 goal definitions. Sample sizes vary across goals due to missing values. Sample sizes for spouse goal attainment are 9764 (weight/BMI), 9031 (aerobic exercise), 9655 (strength training), 9588 (sleep), 9469 (alcohol use – risky drinking), and 9762 (tobacco use – smoking). Sample sizes for service member goal attainment are 9814 (weight/BMI), 9394 (aerobic exercise), 9662 (strength training), 9756 (sleep), 9260 (alcohol use – risky drinking), and 9475 (tobacco use – smoking.). Footnote: ^a^Healthy People is a national study conducted by the Centers for Disease Control and Prevention. Years of data for Healthy People 2020 range from 2005 to 2015

Figure 1 shows HP2020 goal attainment by military spouses and service members. Overall, the majority of spouses and service members met each of the HP2020 goals analyzed (as defined in Table 1). More than half of the responding spouses met the goals related to aerobic exercise (69.1%), sleep (60.7%), alcohol use (75.7%), and tobacco use (79.4%). Only 44.2% of spouses met the healthy weight/BMI goal: 3.0% were underweight, 29.1% were overweight, and 23.7% were obese. Only 42.6% of spouses met the strength training goal of 2 or more days a week. More than half of the responding service members met the goals related to aerobic exercise (78.3%), strength training (68.5%), alcohol use (65.1%), and tobacco use (67.4%). Only 33.7% of service members met the healthy weight/BMI goal and only 41% met the sleep goal. Goal attainment was more prevalent for spouses in weight, sleep, alcohol use, and tobacco use, and more prevalent for service members in aerobic exercise and strength training. Table 3 shows the pairwise agreement of couples with respect to each of the health indicators. All show modest agreement, with concordance percentages ranging from 51 to 73% and kappa coefficients ranging from 0.06 to 0.31 (all *p* < 0.001); dyadic concordance was strongest for alcohol and tobacco use goals.

**TABLE 3:** Concordance of spouse pairs with respect to meeting Healthy People 2020 goals in six domains

	Weight (BMI)		Aerobic Exercise^b^		Strength Training^c^		Sleep		Alcohol Use		Tobacco Use	
	N^a^	% (95% CI)	N^a^	% (95% CI)	N^a^	% (95% CI)	N^a^	% (95% CI)	N^a^	% (95% CI)	N^a^	% (95% CI)
Both Spouse and Service member met HP2020 goal	1876	16.4 (15.4–17.3)	4983	55.9 (54.4–57.4)	3011	31.3 (30–32.6)	3187	27.4 (26.2–28.6)	5162	53.9 (52.5–55.4)	6261	59.7 (58.3–61.1)
Spouse met goal; service member did not meet goal	2822	28.1 (26.8–29.3)	1119	13.3 (12.4–14.3)	1081	11.5 (10.6–12.4)	2787	33.3 (31.9–34.7)	1818	22.4 (21.2–23.6)	1497	19.7 (18.6–20.9)
Spouse did not meet goal; service member met goal	1612	17.4 (16.3–18.5)	1883	22.7 (21.5–24)	3577	37.3 (36–38.6)	1418	13.5 (12.6–14.4)	1006	11.3 (10.4–12.3)	625	7.8 (7–8.6)
Neither spouse nor service member met goal	3399	38.1 (36.8–39.5)	628	8.0 (7.2–8.9)	1783	19.9 (18.8–21.1)	2092	25.8 (24.5–27)	911	12.3 (11.3–13.3)	992	12.8 (11.8–13.7)
Kappa		0.06 (0.03–0.08)		0.08 (0.04–0.11)		0.07 (0.05–0.10)		0.10 (0.07–0.12)		0.20 (0.17–0.23)		0.31 (0.28–0.34)

^a^N’s are unweighted and do not consistently add up to 9872 because of missing data. The number missing ranges from 163 (weight) to 1259 (aerobic exercise)

^b^A total of 927 spouses and 668 service members stated that they did not or could not physically do any aerobic exercise. They were coded as not meeting the aerobic exercise goal.

^c^A total of 4720 spouses and 2125 service members stated that they did not or could not physically do strength training. They were coded as not meeting the strength training goal.

### Multivariable analyses of health behaviors

The multivariable analyses regressing spouse health outcomes on support and military life stressors are shown in Table 4. Of the 6 social and military support independent variables examined, only 2 were statistically significantly associated with any of the HP2020 goals analyzed. Spouses reporting being more bothered by having no one to turn to were significantly less likely to achieve the HP2020 healthy BMI, sleep, risky drinking, and current smoking goals. More specifically, a 1-unit increase in the degree to which spouses were bothered (e.g., from “Bothered a little” to “Bothered a lot”) was associated with a 27% decrease in the odds of meeting the healthy weight/BMI goal, a 32% decrease in the odds of meeting the sleep goal, a 31% decrease in the odds of meeting the risky drinking goal, and a 37% decrease in the odds of meeting the smoking goal. A greater level of perceived support by the military to help the spouse and their family was associated with a higher likelihood of the spouse meeting the sleep goal. Specifically, a 1-unit increase in perceived support from the military to help the spouse and their family (e.g., from “Fair” to “Good”) was associated with a 21% increase in the odds of the spouse meeting the sleep goal.

**Table 4:** Multivariable Logistic Regression Models for Spouse Attainment of Each of Six Healthy People 2020 Goals

Domain	Weight	Aerobic Exercise	Strength Training	Sleep	Alcohol Use	Tobacco Use
Measure	Met HP2020 BMI Goal	Met HP2020 Exercise Minutes Goal	Met HP020 Strength Training Goal	Met HP2020 Sleep Goal	Met HP2020 Risky Drinking Goal	Met HP2020 Smoking Goal
	(*n* = 9105)	(*n* = 8451)	(*n* = 9013)	(*n* = 8981)	(*n* = 8867)	(*n* = 9124)
	Adjusted Odds Ratios (95% Confidence Intervals)					
Spouse demographic characteristics						
Female	**2.65 (2.04–3.43)**	1.07 (0.81–1.42)	**0.60 (0.47–0.76)**	1.03 (0.80–1.31)	**1.45 (1.12–1.88)**	**2.71 (2.03–3.61)**
Age group						
17–24 years	**Ref**	**Ref**	**Ref**	**Ref**	**Ref**	**Ref**
25–34 years	**0.86 (0.74–1.00)**	0.87 (0.74–1.03)	1.05 (0.90–1.22)	0.89 (0.76–1.04)	**1.21 (1.02–1.44)**	0.84 (0.70–1.01)
35+ years	**0.59 (0.46–0.75)**	**0.58 (0.45–0.76)**	**0.70 (0.55–0.90)**	0.81 (0.64–1.04)	**1.66 (1.21–2.29)**	0.98 (0.72–1.35)
Race/ethnicity						
White non-Hispanic	**Ref**	**Ref**	**Ref**	**Ref**	**Ref**	**Ref**
Black non-Hispanic	**0.64 (0.46–0.88)**	**0.70 (0.51–0.96)**	0.96 (0.72–1.29)	**0.72 (0.53–0.97)**	1.46 (1.00–2.13)	**2.13 (1.42–3.19)**
Other	1.06 (0.91–1.23)	**0.81 (0.68–0.96)**	1.08 (0.92–1.26)	1.00 (0.85–1.18)	**1.50 (1.23–1.82)**	**1.93 (1.54–2.42)**
Educational attainment						
High school graduate, GED or less	**Ref**	**Ref**	**Ref**	**Ref**	**Ref**	**Ref**
Some college/Associates degree	1.03 (0.86–1.24)	**1.24 (1.02–1.52)**	**1.37 (1.13–1.67)**	0.88 (0.73–1.07)	**1.26 (1.01–1.57)**	**1.36 (1.11–1.66)**
Bachelor’s degree or higher	**1.33 (1.08–1.64)**	**1.41 (1.12–1.78)**	**1.51 (1.21–1.89)**	1.11 (0.89–1.39)	**1.55 (1.21–1.97)**	**3.54 (2.72–4.63)**
Employment status						
Full-time	**Ref**	**Ref**	**Ref**	**Ref**	**Ref**	**Ref**
Part-time	1.06 (0.86–1.30)	1.03 (0.82–1.29)	1.11 (0.90–1.36)	1.19 (0.96–1.49)	1.04 (0.82–1.30)	1.16 (0.89–1.51)
Unemployed	0.96 (0.77–1.19)	0.85 (0.68–1.07)	0.93 (0.75–1.14)	1.07 (0.86–1.33)	1.08 (0.86–1.38)	0.91 (0.70–1.17)
Homemaker/student	1.03 (0.87–1.22)	0.99 (0.82–1.21)	0.87 (0.74–1.03)	1.07 (0.90–1.28)	**1.61 (1.31–1.98)**	1.05 (0.85–1.31)
Household income						
Less than $25,000	**Ref**	**Ref**	**Ref**	**Ref**	**Ref**	**Ref**
$25,000–$49,999	1.04 (0.86–1.27)	1.22 (0.99–1.50)	0.90 (0.74–1.09)	1.11 (0.92–1.36)	0.93 (0.74–1.17)	0.93 (0.74–1.18)
$50,000–$74,999	1.17 (0.93–1.45)	**1.37 (1.07–1.75)**	1.06 (0.85–1.33)	1.10 (0.87–1.38)	0.83 (0.63–1.08)	1.00 (0.75–1.32)
$75,000 or more	**1.55 (1.18–2.06)**	1.30 (0.96–1.75)	**1.43 (1.09–1.87)**	1.21 (0.91–1.61)	0.78 (0.57–1.07)	1.09 (0.75–1.58)
Number of children						
0	**Ref**	**Ref**	**Ref**	**Ref**	**Ref**	**Ref**
1	**0.85 (0.73–0.99)**	**0.77 (0.64–0.91)**	**0.78 (0.67–0.91)**	**0.64 (0.55–0.76)**	**1.88 (1.57–2.25)**	**1.30 (1.06–1.59)**
2+	**0.76 (0.65–0.89)**	0.98 (0.82–1.17)	0.86 (0.73–1.01)	**0.55 (0.47–0.65)**	**1.80 (1.50–2.17)**	1.00 (0.83–1.21)
Spouse military service						
Never	**Ref**	**Ref**	**Ref**	**Ref**	**Ref**	**Ref**
Former	0.96 (0.76–1.21)	1.08 (0.84–1.39)	1.01 (0.80–1.26)	**0.76 (0.61–0.96)**	**0.75 (0.59–0.97)**	0.90 (0.68–1.20)
Current	1.15 (0.89–1.50)	**2.63 (1.86–3.71)**	**3.03 (2.31–3.97)**	**0.71 (0.55–0.93)**	1.13 (0.85–1.51)	1.17 (0.84–1.61)
Service member military characteristics						
Reserve/National Guard (vs active duty)	0.97 (0.82–1.13)	0.81 (0.68–0.96)	**0.77 (0.66–0.91)**	0.95 (0.80–1.12)	1.15 (0.95–1.40)	1.23 (1.00–1.51)
Pay grade – officer (vs enlisted)	**1.73 (1.44–2.08)**	**1.27 (1.04–1.56)**	0.92 (0.77–1.10)	**1.26 (1.04–1.52)**	1.07 (0.86–1.32)	**3.29 (2.43–4.45)**
Service branch						
Army	**Ref**	**Ref**	**Ref**	**Ref**	**Ref**	**Ref**
Air Force	1.06 (0.91–1.24)	1.12 (0.93–1.34)	1.04 (0.89–1.21)	**1.24 (1.05–1.46)**	**1.32 (1.08–1.61)**	**1.59 (1.28–1.97)**
Coast Guard	**1.79 (1.27–2.53)**	1.38 (0.93–2.03)	1.27 (0.91–1.79)	1.40 (0.97–2.00)	0.77 (0.52–1.14)	1.36 (0.85–2.17)
Marine Corps	1.15 (0.94–1.39)	1.15 (0.91–1.44)	0.88 (0.72–1.07)	1.12 (0.91–1.37)	**0.77 (0.61–0.96)**	**1.70 (1.30–2.21)**
Navy	1.17 (0.97–1.42)	1.03 (0.83–1.26)	1.04 (0.86–1.25)	1.21 (1.00–1.47)	1.09 (0.87–1.36)	**1.44 (1.12–1.86)**
Military stress						
Deployment stress (0–4)	**1.08 (1.01–1.16)**	1.05 (0.97–1.14)	1.06 (0.99–1.15)	0.95 (0.88–1.02)	1.00 (0.92–1.09)	1.00 (0.91–1.10)
Injury stress (0–4)	1.01 (0.94–1.08)	0.98 (0.91–1.05)	1.01 (0.94–1.08)	1.04 (0.97–1.12)	0.98 (0.91–1.07)	0.97 (0.89–1.05)
Family stress (0–4)	0.98 (0.91–1.06)	0.98 (0.90–1.07)	1.03 (0.96–1.11)	0.95 (0.88–1.03)	0.93 (0.85–1.01)	1.02 (0.93–1.12)
Permanent change of stations (PCS) stress (0–4)	0.99 (0.95–1.04)	1.02 (0.97–1.08)	1.04 (0.99–1.09)	1.00 (0.96–1.05)	0.98 (0.93–1.03)	1.04 (0.98–1.11)
Social and military support						
How much spouse is bothered by having no one to turn to (1–3)	**0.73 (0.65–0.82)**	1.03 (0.91–1.17)	0.90 (0.80–1.01)	**0.68 (0.61–0.77)**	**0.69 (0.61–0.78)**	**0.63 (0.54–0.72)**
Spouse has someone to turn to for suggestions on dealing with personal problems (0–4)	1.03 (0.92–1.14)	1.12 (0.99–1.27)	1.07 (0.96–1.19)	1.07 (0.95–1.20)	1.08 (0.95–1.22)	1.06 (0.92–1.22)
Spouse has someone to tell them honestly how they’re handling problems (0–4)	1.00 (0.90–1.10)	1.05 (0.94–1.17)	1.04 (0.93–1.15)	1.03 (0.93–1.15)	0.94 (0.84–1.06)	0.88 (0.77–1.01)
Support from family or friends (0–4)	0.99 (0.93–1.05)	1.02 (0.96–1.10)	1.01 (0.95–1.07)	1.02 (0.96–1.09)	1.04 (0.98–1.12)	0.98 (0.91–1.06)
Military efforts to help spouse and family (0–4)	1.05 (0.96–1.14)	0.97 (0.89–1.06)	1.07 (0.99–1.16)	**1.21 (1.12–1.32)**	1.03 (0.94–1.14)	1.09 (0.98–1.21)
Military efforts to help service member (0–4)	0.98 (0.90–1.06)	1.02 (0.94–1.12)	0.98 (0.91–1.06)	0.93 (0.86–1.01)	0.98 (0.89–1.08)	0.94 (0.85–1.04)

*Note*. Family Study weights are used in all models [24]. All social and military support independent variables are multi-category ordinal variables that are used as continuous measures in the models. The odds ratio reported for each of these variables corresponds to a 1-unit increase in the independent variable. The numbers in parentheses refer to the scoring of the independent variables (see Table 2 for details). Significant effects at the *P* < .05 value are bolded

Of the 4 types of military stressors included in our models as predictors (deployment, injury, family, and PCS), only deployment-related stressors were marginally significantly associated with any of the spouse health indicators. A one-unit increase in stress related to deployment experiences (e.g. from slightly to moderately stressful) was associated with an 8% increase in the odds of spouses meeting the HP2020 BMI goal.

#### Spouse demographic characteristics

Female spouses were much more likely than male spouses to meet the HP2020 goals for weight as well as both alcohol and tobacco use, with more than twice the odds of meeting the weight and tobacco use goals. Female spouses were considerably less likely to meet the strength training goals. Older spouses, and in particular those over age 35, were less likely to meet the healthy weight goal. Those aged 35 and older were also less likely to meet either of the exercise goals but more likely to meet the alcohol use goal. Compared to Whites, Black non-Hispanic spouses were less likely to meet the weight, aerobic exercise, and sleep goals but much more likely to meet the tobacco use goal. Those of other races were also less likely to meet the aerobic exercise goal but more likely to meet both of the substance use goals. Greater educational attainment confers greater likelihood of meeting each of the HP2020 goals, except for sleep for which there was no association. In general those with a college degree had better outcomes than those with some college, who in turn did better than those with no college. In particular, those with at least a bachelor’s degree had 3.5 times the odds of those with no college of refraining from tobacco use. Those identifying as homemakers or students were more likely to meet the alcohol use goal than other employment groups; no other associations between spouse employment status and meeting the HP2020 goals were observed. Spouses with children were less likely to meet the weight, exercise, and sleep goals but more likely to meet the substance use goals. Compared to those with no personal military experience, spouses who were themselves current members of the military were much more likely to meet both exercise goals than non-military spouses; however, they were less likely to meet the sleep goal. Spouses who were formerly in the military were also less likely to meet the HP2020 goal for sleep and also less likely to meet the goal related to risky alcohol use.

#### Service member military characteristics

Spouses of military reserve or National Guard members were less likely to meet the strength training goal, but no other differences with respect to military component were observed. Spouses of officers were more likely to meet the weight and aerobic exercise goals and also had more than three times the odds of meeting the tobacco use goal compared to spouses of enlisted soldiers. Compared to the spouses of service members in the Army, spouses of Air Force members were more likely to meet the sleep goal as well as both substance use goals. Marine spouses were less likely to meet the risky alcohol goal but more likely to not use tobacco. Navy spouses were also more likely than Army spouses to meet the tobacco use goal.

## Discussion

Overall, the majority of military spouses and service members met most of the HP2020 goals analyzed in the study. However, less than half of military spouses met the healthy weight/BMI goal or the strength training goal. Spouses were more likely to achieve healthy weight, sleep, and alcohol and tobacco use goals than were service members, whereas more service members met the aerobic exercise and strength training goals likely due to physical health demands of military service. In addition to comparing military spouses with their partners, it is important to contextualize these results by comparing military spouses with the U.S. adult population. To do so, we compared rates from the current study with the 10-year HP2020 national targets, which represent the aims that the government sets at a population level, acknowledging that these comparisons must be interpreted cautiously due to demographic differences between the target population of the Family Study and the U.S. adult population [30]. A higher proportion of military spouses (44.2%) met the HP2020 healthy weight/BMI goal compared with the national target of 33.9%. Relatedly, fewer military spouses were obese (23.7%) compared with the national obesity target of 30.5%. Sixty-eight percent of military spouses met the HP2020 physical activity objective and 42.6% met the strength training objective, higher than the national targets (47.9 and 24.1%, respectively). The proportion of military spouses meeting the sleep objective (61.3%) was lower than the national target of 70.8%. A comparable proportion of military spouses did not meet goal related to risky drinking, compared with the national target (24.3% vs 25.4%). More military spouses reported currently smoking than the national target (20.6% vs 12.0%). Overall, compared with the HP2020 targets for the entire U.S. adult population, a higher proportion of military spouses met the objectives for healthy weight, obesity, and physical activity than the national targets, while fewer met these targets for sleep and smoking. It is unclear to what extent these differences may be explained by the younger age and other demographic differences between this study’s target population and the adult population as a whole. The Centers for Disease Control and Prevention (CDC) does not provide demographic breakdowns for its population targets.

Multivariable results suggest that social support and perceived support from the military are associated with military spouses’ health behaviors. Specifically, spouses who reported being bothered by not having someone to turn to when they were having a problem were less likely to achieve a healthy weight or sufficient sleep and were more likely to engage in risky alcohol use and to smoke cigarettes. Similarly, when spouses perceived greater efforts by the military to support their families, they were more likely to meet the healthy sleep goal. Research suggests that spouses identify multiple ways their military lifestyle makes it difficult to maintain strong social connections, including frequent moves, living far from family and friends, and lack of time [31]. Together, these results suggest providing resources to assist spouses in developing social networks and support, and addressing identified barriers to connectedness, may have broader implications on the overall health and well-being of spouses. Several spouse demographics were associated with health indicators and were controlled for in the multivariable models, including gender, age, ethnicity and education. Additionally, spouses of officers, compared to enlisted personnel, were more likely to meet the weight, aerobic exercise, and tobacco goals and spouses of Air Force members were more likely to meet the sleep and substance use goals compared to Army spouses.

Based on our results, it is clear that service member and spouse health behaviors are associated and likely influence one another bidirectionally. These findings suggest that enhanced support and program for either or both partners may assist the couple and improve family readiness. Although there are many existing social support and military health promotion programs available, most target service members rather than military spouses, and there is a lack of unified family resource programs [32]. Existing family programs include Military OneSource, which disseminates information on all military family health resources; Operation Live Well, an initiative to improve health and wellness for the entire defense community, and its Healthy Base Initiative targeting service members, DoD civilians, and their families. The U.S. Army Public Health Center Performance Triad includes a specific resource page for spouses with educational materials and social media resources that aim to improve sleep, physical activity, and nutrition and operate the Army Wellness Centers that are available to military spouses. Building upon these existing military health promotion and social support programs to be more accessible and targeted to military spouses could have direct implications for increasing positive health behaviors synergistically among service members and their spouses.

Military experiences associated with injury, PCS moves, and family stress were not significantly associated with the health behavior outcomes in this study. Interestingly, having more stressful experiences related to deployment was associated with a greater likelihood of having a healthy BMI. This finding is inconsistent with previous research. For example, Fish and colleagues (2014) found that deployment has no relationship with healthy weight, but that male Army spouses were more likely to be obese or overweight than female spouses [8]. Padden and colleagues (2011) found that deployment was associated with poorer dietary behaviors [33].In the current study, deployment-related stress was associated with only a single health outcome, healthy weight/BMI, and that relationship was fairly weak, suggesting that deployment-related stress and PCS moves may not have a strong or consistent influence across health behaviors. As Family Study follow-up data for this longitudinal effort become available, it will be possible to further investigate these relationships prospectively. Future studies might assess if other military-related stress influences health indicators over time and if there are directional effects in terms of behavioral influence between the spouses in meeting the goals, and if they are stronger from the service member to the spouse or vice versa. Such studies could inform the most effective points of prevention and intervention for military families. Future longitudinal research could also assess more comprehensive bio-psycho-social models predicting health outcomes for military spouses to distinguish the strongest influencing factors, including behavioral health predictors such as Posttraumatic Stress Disorder which has been linked to health outcomes in various studies [34–36].

### Limitations and strengths

There are a number of limitations to this study that should be considered. The data are largely based on self-report which can be vulnerable to bias, and it would be ideal to also have observational or medical data to validate the health outcomes. However, CDC measures on the national health objectives are also based on self-report, making these measures more comparable. The reports are also retrospective, meaning that spouses and service members reported on their health behaviors over a specified time period (e.g., the last month) and may have experienced poor or biased recall. Additionally, there are missing data, particularly on the item related to PCS stress. Finally, only married couples of the opposite sex were included in the study, thus the results may not generalize to lesbian, gay, bisexual, and transgendered spouses or to single-parent households. Despite these limitations, the study has considerable strengths. The cohort includes a representative sample of young military couples across service branches and components, including active duty, military Reserve, and National Guard participants. The study cohort also includes both female and male military spouses and data acquired from both the spouse and service member. The constructed self-report health measures also closely align with the HP2020 objectives, enabling comparisons of military spouses’ health behaviors and national benchmarks.

## Conclusions

Using the HP2020 objectives as a framework for identifying key health behaviors and benchmarks, this study identified factors, including military-specific experiences that may contribute to physical health behaviors and outcomes among military spouses. The findings provide important insights that could help inform health promotion programs for military families, improve force readiness and retention, and enhance the well-being of military families. The study also offers a unique contribution to the HP2020 efforts by revealing the proportion of military spouses, a large and important segment of the population, who meet several key health objectives.

The spouses in this study face similar challenges in maintaining a healthy lifestyle as individuals in the broader civilian population, but they must also navigate additional stressors related to their role as part of a military dyad. These stressors may include having a spouse who is deployed, not having a strong social support system, and not feeling supported by the military. It is important that these spouses are provided with the support services and programs to help them maintain and improve their health behaviors and improve the overall health and well-being of U.S. military personnel.

## Data Availability

The datasets analyzed during the current study are not publicly available; deidentified data are available upon the establishment of a Department of Defense data use agreement.

## Abbreviations

APHC: Army Public Health Center
BMI: Body mass index
CDC: Centers for Disease Control and Prevention
DoD: Department of Defense
Family Study: Millennium Cohort Family Study
HP: Healthy people
PCS: Permanent change of station
